# Effects of a warming trend on cool climate viticulture in Michigan, USA

**DOI:** 10.1186/s40064-016-2777-1

**Published:** 2016-07-19

**Authors:** Steven R. Schultze, Paolo Sabbatini, Lifeng Luo

**Affiliations:** Department of Geography, Michigan State University, East Lansing, MI 48824 USA; Department of Horticulture, Michigan State University, East Lansing, MI 48824 USA; Center for Global Change and Earth Observations, Michigan State University, East Lansing, MI 48824 USA; Department of Earth Sciences, University of South Alabama, Mobile, AL 36688 USA; 5871 USA North Drive, Life Sciences Classroom Building (LSCB), Room 136, Mobile, AL 36688-0002 USA

**Keywords:** Climate change, Agriculture, Landscape change, Great Lakes, Viticulture, Climate model projections

## Abstract

Historically, Michigan’s climate had mainly three challenges for grape production: growing season temperatures were too low, the growing season was too short and there was too much rain near harvest. However, climate change in the past decades has led to a vastly different landscape that is evolving to meet the new climate. Recently, there has been a significant move from *Vitis labrusca* (North American) grape plantings to *Vitis vinifera* (wine grapes) as a consequence of Michigan’s shifting climate. The goal of this study was to analyze the historical shift in climate and its potential future impact on the grape industry. We obtained data climate model projection data from two greenhouse gas (GHG) emission scenarios. First, a multi-linear regression model was built to predict future grape yields (t/ac) using data from the climate model projections. Second, trends in the severity of the three challenges (temperature, season length, precipitation timing) were analyzed. In both GHG scenarios grape yields are seen to improve, but to different extents. The improvement is likely a response to warmer season temperatures canceling out losses to early season frost. Model projections recommend that Michigan’s future climate will be more accommodating for all varieties of grapes. This suggests that grape production will continue to grow, but the landscape will continue to evolve with more emphasis on varieties that are more climatically sensitive to cold temperatures. Climate change has greatly affected Michigan’s viticultural landscape, and will continue to do so in the coming decades.

## Background

From a climate perspective, Michigan is considered a “cool-cold” climate viticulture region of the world: cool is referred to the summer and cold is referred to the winter temperature (Gladstones 1992; Zabadal and Andresen 1997). This classification is due to a combination of climate challenges that the grape growing industry has historically encountered. The first issue is the average growing season temperature (GST). Michigan’s average GST in the 1950s and 1960s was 14.1 °C in the northwest corner of the lower peninsula and 16.5 °C in the southwest corner of the State. Both areas experienced summers with appreciable variability on a yearly basis, with a 0.19 and 0.21 °C standard deviation, respectively, in temperature during the 20-year period. Michigan’s two potential regions for *Vitis vinifera* production were generally too cool, as any warm years would occur unreliably. The second issue centered on the distribution of monthly rainfall. Michigan’s west coast is classified as a *Dfa* Köppen climate classification (Köppen 1900; Geiger 1965), with consistent precipitation year-round. However, the peak of precipitation occurs in the months between August and October, which coincide with veraison (August), the vine phenological stage where the fruit begins to ripe and harvest (September and October). Rainfall after veraison and during harvest increases yield loss due to disease and poor fruit technological maturity (Gladstones 1992; Zhuang et al. 2014). Consequently, Michigan’s monthly rainfall distribution was considered to be a negative factor in the production of *vinifera* grapes. In addition to temperature and precipitation concerns, Michigan’s growing season was considered relatively short for several grape varieties commercially important in US (e.g. Cabernet Sauvignon, Cabernet Franc, Merlot). As of the early 1970s, Michigan’s growing season from budburst to first fall frost was approximately 160 days (Schultze et al. 2014). The grape growing season was limited by late bud-bursts, potential hazardous frosts in the spring and an unreliable timing of the first fall frosts ranging from as early as 13 September to as late as 30 October (Jasper and Holloway, personal communication, August 1, 2012). Early and late season frosts can be particularly damaging to vines and are thus of concern in cooler climates. Exposure to air temperatures at or below −1 °C can significantly damage buds in the early season (Zabadal and Andresen 1997) and expose fruit to risk at the end of the growing season (Molitor et al. 2014). Data are limited to the southwest of Michigan, and we assume that the growing season in the northwest (not as extensively measured for grape production as in the southwest) was shorter due to even later bud-bursts and earlier fall frosts.

When examining the three major concerns of the region were that Michigan was too cool, was too wet at the wrong time and had a short season, it is logical that grape production was limited to *Vitis labrusca*, a cold-hearty, North American variety mainly used in production of juice and jams. Further, it is remarkable that with that knowledge, *vinifera* production even began in the late 1960s (Hathaway and Kegerreis 2010). However, since *vinifera*’s initial plantings, the landscape of Michigan’s grape growing industry has shifted extensively and has grown into a considerable industry in a relatively short amount of time. Part of the reason this has occurred is almost certainly due to Michigan’s climate becoming more favorable for *vinifera* production in recent decades (Schultze et al. 2014).

The climate in the region of the Great Lakes has experienced warming commensurate with the global trend in higher temperatures with the region experiencing an approximate 1.0 °C increase in temperatures since 1980 (Andresen et al. 2012). Schultze et al. (2014) found the shift in southwest Michigan’s climate since 1980 contributed to a 3.7 growing degree day (GDD) per year increase (base 10 °C) in southwest Michigan; a trend likely to continue into the near future. Research in other cool climate viticultural areas such as the Alsace region of France (Duchêne and Schneider 2005) southern Canada (Jones 2012), northern Europe (Molitor et al. 2014), found that recent climate change has altered the viticultural landscape with respect to varieties that can be accommodated in their respective regions. This fits in with the general theme of climate change greatly affecting the traditional concept of “terroir,” as an area’s climate can no longer be treated as a static entity (Pfister 1988; Gladstones 1992; Jones et al. 2005, 2010; van Leeuwen et al. 2004; Seguin and de Cortazar 2005).

Schultze et al. (2014) also found that GDD accumulations in Michigan between April and October in the 1950s and 1960s were typically between 1300 and 1500 units, which is considerably lower than the long term mean from 1980 to 2011 of 1628. It should be noted that *V. vinifera* production in Michigan did not begin until the late 1960s, and did not expand beyond a few small plots until the 1980s. According to the USDA, from 2000 to 2011, Michigan underwent a nearly 300 % increase in *vinifera* acreage (USDA-NASS 2012). GDD accumulations in the first decade of the 2000s were typically in the >1600 range, and never below 1400 units. This reflects the fact that Michigan’s warming climate is becoming more conducive for *vinifera* production. Michigan’s ability to go from incapable of supporting *vinifera* production to becoming a region of considerable production of wine grapes places Michigan in a “zone of transition”; from being able to support primarily one species of grape (juice grapes, *V. labrusca*) to being able to accommodate a wide range of wine grape (*V. vinifera*) varieties.

The goal of this research was to explore the possible direct and indirect effects of climate change on grape production in Michigan over the next decades up to the end of the twenty-first century. A previous study (Schultze et al. 2014) was focused solely on the southwest portion of Michigan due to the availability of long-term yield data acquired from the National Grape Co-operative (Jasper and Holloway, personal communication, August 1, 2012). This study will discuss potential implications on vine yield for the southwest region of Michigan, but climate projections will still be addressed for the northwest, as it is likely to continue to be an important part of Michigan’s wine grape industry. The aforementioned three primary obstacles will be addressed for the Michigan’s potential *vinifera* industry and how shifts in climate will help the region overcome those problems. This work will use data obtained from a number of sources including; (1) the National Grape Cooperative, (2) the Coupled Model Intercomparison Project Phase 5 (CMIP5) downscaled version of the NASA Earth Exchange Downscaled Project-30 Arc Second (NEXDCP-30) dataset, (3) Michigan State University’s Enviro-Weather Mesoscale network and (4) National Climatic Data Center to evaluate potential trends in Michigan climate. Climate model data has been used before in viticulture related studies (Bindi et al. 1997; Jones et al. 2005; Jones and Goodrich 2007; Santos et al. 2011). However, as of the time of the initial study, CMIP5 data and the NEXDCP-30 data sources had not been utilized in a viticultural landscape study in any such way. By combining these sources, issues such as season length, GST, rainfall distributions, potential yields and potential new varieties of *vinifera* from 2012 to 2099 can be addressed. This study focuses on climate projection data for both the southwest and northwest of Michigan.

## Methods

### Site description

**Fig. 1:**
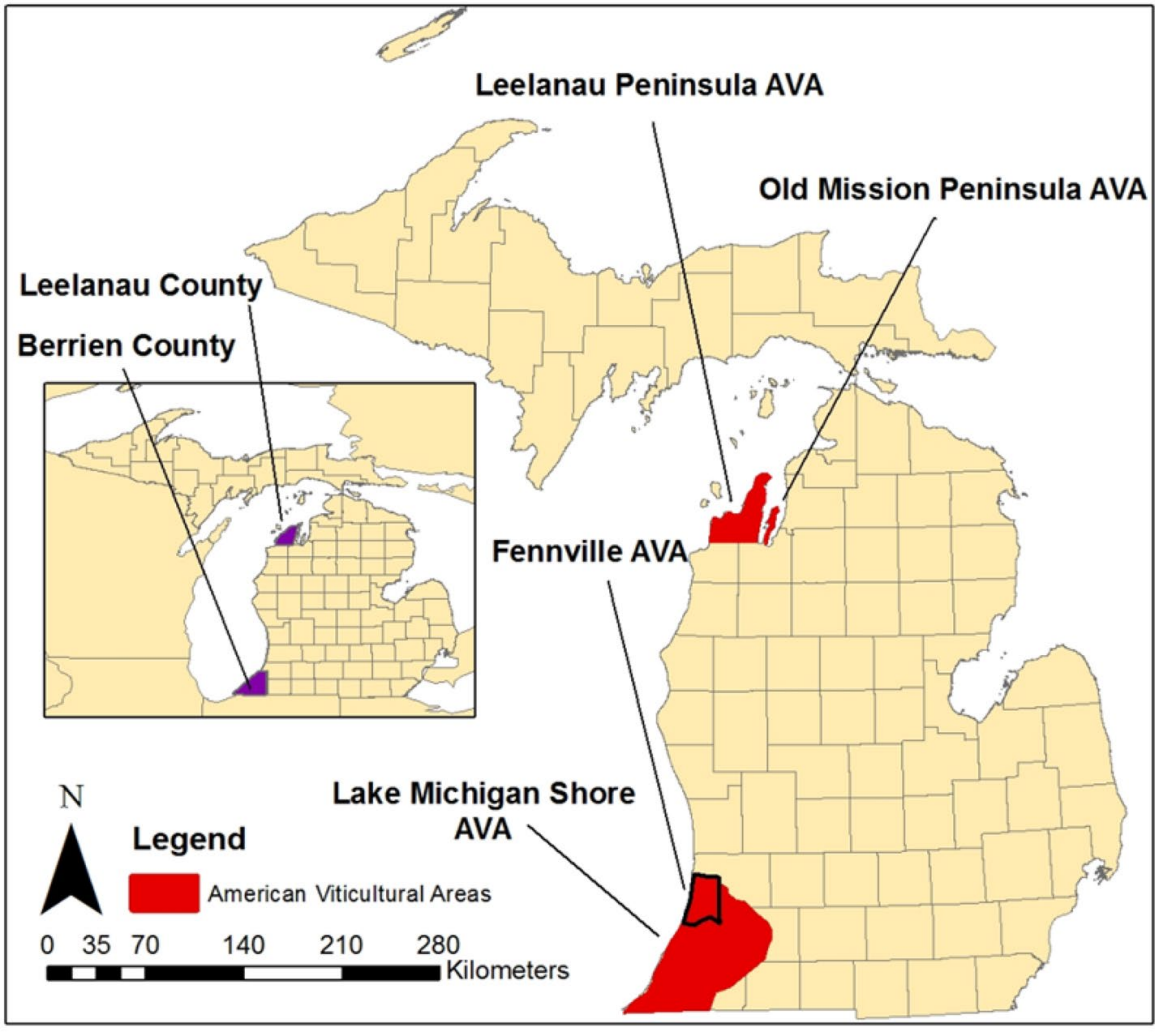
Map of Michigan’s American viticultural areas (AVAs). Leelanau and Old Mission Peninsula AVAs will be referred to as “Northwest Michigan” and Fennville and Lake Michigan Shore AVAs will be referred to as “Southwest Michigan” in this paper

The primary grape producing areas in Michigan are located in the west coast of the lower peninsula spread throughout four American Viticultural Areas, which are geographically defined areas that designate where grapes are grown based on local geographic features. There are four AVAs within the state of Michigan, two of which are located in the southwest corner (Fennville, Lake Michigan Shore) and two in the northwest portion of the lower peninsula (Leelanau, Old Mission) (Fig. 1). The reasons for the location near the shores of Lake Michigan are: (a) the climate moderating effects of the Lake and (b) topographic influences, which allow for drainage of cold air during the spring and fall seasons. Growing *vinifera* grapes farther inland or in flat regions in Michigan is not recommended, as spring/fall frosts and harsh winter temperatures can combine to potentially damage or even kill vines (Zabadal and Andresen 1997). These potentially dangerous temperatures still occur in the coastal areas where *vinifera* grapes are grown, but site selection and vineyard management are key to mitigating these potential damages. Thus, large-scale losses are less common in these regions as compared to areas as little as 50 km inland. However, in areas close enough to the close to experience the temperature moderating effects of the lakes, growers have success growing a number of *V. vinifera* types including Cabernet Franc, Chardonnay, Gewurztraminer, Pinot Noir and Riesling among other *vinifera* and hybrid varieties.

Southwest Michigan, located around 41° North latitude, is classified as a *Dfa*, humid continental climate in the Köppen Climate Classification system (Köppen 1900; Geiger 1965). Northwest Michigan, located around 45° north latitude, is classified as a *Dfb* climate with shorter summers and colder winters than areas to the south. However, the small areas that are located within the Leelanau and Old Mission AVAs are areas located on peninsulas (the AVAs are named for their respective peninsula) in Lake Michigan and Grand Traverse Bay. The ability to grow *vinifera* grapes in these regions is due almost entirely to the presence of favorable microclimates where the temperature during fall, winter and spring are much warmer than surrounding areas. It is likely that the microclimates in the northwest are much more similar to the *Dfa* climates found in the southwest Michigan AVAs. A combination of microclimates and the close proximity of the Lake help to limit temperature extremes compared to areas farther inland due to consistent lake and land breezes (Moroz 1967).

### Data collection

This research relies on future climate projections from the fifth phase of the Coupled Model Intercomparison Project (CMIP5) suite of climate models. CMIP5 was developed to answer the many questions posed by the Intergovernmental Panel on Climate Change (IPCC)’s fourth assessment report (Solomon 2007). Among the many potential improvements to the experiments, one group of hypothetical scenarios is of particular interest to our research. Included in the model experiments were four different, transient greenhouse gas (GHG) scenarios wherein the amount of global emissions of GHGs followed different potential cases. These “representative concentration pathways” (RCP) scenarios would project global GHG emissions in the coming decades using different econometric and social models where the number following “RCP” represents the increase in radiative forcing values (measured in W/m^2^) by the year 2100 relative to pre-industrial values (Van Vuuren et al. 2011). In one such scenario (RCP4.5), GHG emissions are reduced on a global scale at a certain point in the future on the assumption of policy action by global leaders. In another scenario (RCP8.5), very little to no action is taken. As such, these scenarios involve different reactions by global temperatures to these hypothetical GHG emission scenarios (Taylor et al. 2012).

The results of many of the CMIP5 models were released in 2013. However, one of the limitations of such a large undertaking when creating such a large model projection dataset is that model resolution has to be sacrificed limited by computational power, storage space among other factors. Most model runs in the CMIP5 models have a resolution of approximately 100 km. Projections at such a resolution is useful for continental to global scale studies, but problematic for regional scale applications. This problem, according to CMIP5’s executive summary, presents an issue with point observations and with the spatial areal issue, in that a spatially averaged value (one grid-cell) is not representative of a point observation within the grid-cell (Taylor et al. 2012). One method to manage this concern is through the downscaling of grid cells from a lower resolution to a higher resolution. The NASA Earth Exchange Downscaled Project (NEX-DCP30) is one project that downscaled a number of CMIP5 model runs down to a resolution of 800 m for the entire contiguous 48 United States. The dataset was created using the bias correction spatial disaggregation (BCSD) methodology established in Wood et al. (2004). This methodology takes in climate model data with larger pixel resolutions and time-steps and uses real-world observations and interpolation methods to bring the data down to finer resolution in both the spatial and temporal dimensions. NEX-DCP30 allows users to use future climate projections to perform environmental analysis at a manageable resolution. These projections are based on data obtained from the Parameter-elevation Relationships on Independent Slopes Model (PRISM) temperature data and this data transitions seamlessly into 32 different CMIP5 models until the year 2100 at a monthly time step for three variables: temperature max (Tmax), temperature min (Tmin) and precipitation. This downscaled analysis of the climate models allows for a high-resolution analysis of future trends. It is of particular interest in an area like the Great Lakes Region, where the land–water interface is highly difficult to resolve in models where the resolution is bigger than 50 km.

The NEX-DCP30 downscaled data was compiled from the National Climate Change Viewer (http://www.usgs.gov/climate_landuse/clu_rd/apps/nccv_viewer.asp), managed by the United States Geological Survey (USGS). The downscaled data was downloaded on a county scale and averaged over the county in focus. The two counties focused on in this study were Berrien county (southwest MI) and Leelanau county (northwest MI). These counties, combined, account for a significant portion of Michigan’s *vinifera* production and are likely to expand in acreage in the coming decades, potentially following the near 300 % trend in *vinifera* acreage growth from 2000 to 2011 (USDA-NASS 2012). The dataset, downscaled to a resolution of 800 m and then averaged over the county area, does introduce uncertainty as the data was downscaled from the much larger climate model scaled projections. However, such a fine resolution is needed for studies where microclimates are a part of the study and is crucial in a region like the Great Lakes, where the land–water interaction is either idealized or roughly estimated due to the coarse resolutions of the models. In fact, viticultural studies at large could benefit greatly from high-resolution climate model data even in areas where the water-land interaction is less important. The western United States is one such area (Jones et al. 2010) with its heavy topography, or in Portugal, an area with a multitude of microclimates in even a small area (Fraga et al. 2015).

Averaging the NEX-DCP30 data over a county is also reflective of the dataset for yields used in this study. The data obtained from the National Grape Co-operative was taken as the average from 25 plots from around the southwest portion of Michigan. The age of the vineyards was between 15 and 30 years old. Own-rooted Concord vines were trained to 1.8-m high bilateral cordons with a north–south row orientation. Vines were spaced 2.38 m in row and 3.05 m between row (Jasper and Holloway, personal communication, August 1, 2012). The use of Concord vines, rather than *vinifera* vines, is due to the robust size of the Concord vine phenology dataset. Data for *V. labrusca* grapes in Michigan exists for several decades, while the equivalent data for *V. vinifera* does not exist for nearly the same length of time as *vinifera* phenology tracking in Michigan has only been occurring in the recent past.

### Experimental design

In order to describe how the continual warming trend has affected and will continue to affect Michigan’s wine grape industry, it was necessary to use data from historical sources in conjunction with future projections. First, temperatures (max, min and mean) were calculated for the growing season (1 April–31 October) for both regions considered in the study. The NEXDCP30 dataset (future projections) has data obtained from 32 model runs plus one ensemble mean of all models run in the RCP4.5 and RCP8.5 GHG emission scenarios. However, these model simulations begin in the year 1950. The historical data from these models was developed by incorporating PRISM temperature and precipitation data when the creators were using the BCSD method of downscaling (Wood et al. 2004). This downscaled data was used as historical climate in southwest and northwest Michigan in this study from 1950 to 2005. From 2006 to 2099, there were 32 different model runs for each RCP scenario, and there was one ensemble mean for all models. This historical and future projection data was then used as the input for the analysis in this paper.

This analysis includes a multilinear regression based on past climate and grape yields in order to predict future trends for grapes. Similar concepts in methodology for the application of future climate projections into statistical models for estimating past and future grape yields can be found in Lobell et al. (2006), Santos et al. (2011), Fraga et al. (2014). This multilinear regression is limited only to the southwest of Michigan, as that is where the yield data has been recorded for decades by the National Grape Cooperative. This regression calculates potential yield in future years based on both historical data and monthly and seasonal climate projections in the RCP4.5 and RCP8.5 scenarios based on five variables: average GST, growing degree day totals (GDD), potential early season frost occurrence (Frost), total season precipitation (PPT) and early season GDD accumulation (eGDD). NEXDCP30 data was used to directly obtain two data sources (GST, PPT, eGDD) and to indirectly obtain the other variables using regressions (Frost, GDD).

1 $$\begin{aligned} \frac{T}{acre} & = - 27.662 + (0.0778 \times PPT) + (1.578 \times GST) - (0.542 \times Frost) \\ & \quad + (0.0017 \times GDD) - (0.022 \times eGDD) \\ \end{aligned}$$

Regression model for approximation of potential yield of *V. labrusca* where T/acre = tons per acre of production, PPT = total season precipitation, GST = average growing season temperature, GDD = growing degree day total, Frost = potential early season frost occurrence, and eGDD = early season GDD accumulation.

This regression is prone to uncertainty due to the data sources (downscaled climate model data), but the goal of using this model is to look at how the RCP4.5 and RCP8.5 scenarios potentially affects grape yields under future scenarios in southwest Michigan.

The study also includes analysis on the region’s changing climate and how it affects the aforementioned three primary concerns for *vinifera* cultivation in Michigan. Future GST, monthly precipitation distributions and season length are all considered under the RCP4.5 and RCP8.5 scenarios in the future out to the year 2099 for southwest and northwest Michigan and the changes are discussed. Finally, there is a discussion of Michigan’s potential future varieties using the future climate projections and known climate thresholds for a number of *vinifera* varieties. This section is meant as a hypothetical scenario for future decades where *vinifera* acreage in Michigan continues to expand and new varieties are considered for the region.

## Results

### Temperature trends

Traditionally, Michigan’s grape growing region is classified as a “cool climate”. However, the average temperatures over the course of two 30-year periods starting in the year 1950 show significant change. In southwest Michigan, GSTs from 1950 to 1979 averaged 16.50 °C. From 1980 to 2009, the growing season average temperature rose 0.55–17.05 °C. In northwest Michigan, a nearly identical warming trend was found. From 1950 to 1979, the average GST was 14.13 °C while from 1980 to 2009, the average temperature was 14.69 °C (increase of 0.56 °C). The average GSTs for both southwest and northwest Michigan suggest that Michigan’s grape growing regions may not necessarily need to be combined as one homogenous region and southwest Michigan is, on the long term average, approximately 2.4 °C warmer than northwest Michigan. From a temperature perspective, these two regions have two different climates. This is represented by the fact that southwest Michigan is a *Dfa* Köppen climate class, while northwest Michigan is a *Dfb* class (Köppen 1900; Geiger 1965). The second issue is that southwest Michigan’s average GSTs should be seen as too warm to be classified as a “cool climate”, at least from the perspective of growing season mean temperature. Michigan’s growing season has traditionally been too short from spring to fall, and Michigan’s winters would certainly classify the region as cool. However, from an average GST perspective, Michigan’s *vinifera* production regions are not homogeneous and may not need to be classified as “cool climate viticulture.”

### Potential future yields

**Fig. 2:**
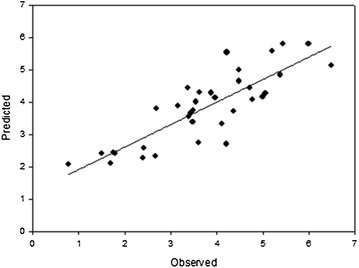
Predicted versus observed results in yields (t/ac) from 1975 to 2011 for southwest Michigan *V. labrusca* (*R* = 0.81, *R* ^2^ = 0.66, MAE = 0.59)

A statistical model was built to illustrate the effects of potential changes in climate on vine production for southwest Michigan. The reason for the model being built for southwest Michigan, and not for northwest Michigan, was that long-term historical yield data has only been compiled for the southwest corner of the state. The statistical model, a multi-linear regression, had an input of five independent X variables (precipitation, average GST, potential frost occurrence, GDD accumulation and early season GDD Accumulation) as predictors of yield (t/ac). The model was found to have good accuracy, with a Pearson’s *R* = 0.81 (*R*^2^ = 0.66) and a mean average error of 0.59 in the years from 1975 to 2011. The model was validated by using the cross-two out validation method. Two seasons were removed from the model and predicted using the regression and in both seasons, the error was <0.4 t/ac, indicating good relative accuracy (Fig. 2).

**Fig. 3:**
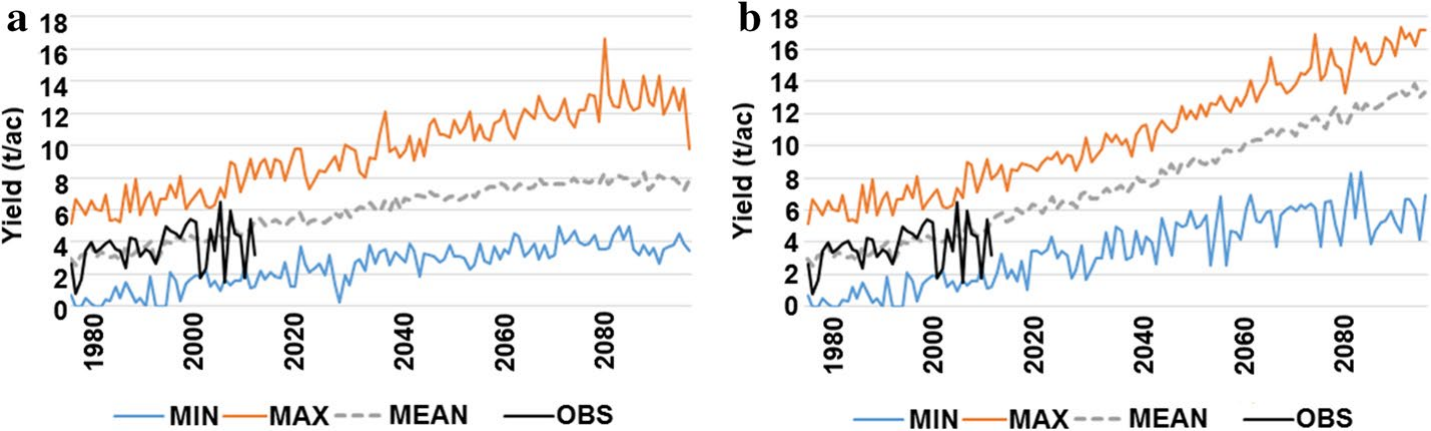
Potential yields for *V. labrusca* grapes based on minimum temperature, maximum temperature, model mean in southwest Michigan from 1975 to 2099 with data from the RCP4.5 greenhouse gas emissions scenarios (**a**) and the RCP8.5 greenhouse gas emissions scenarios (**b**) as well as observed yields

Using the multilinear regression built from historical data, future climate projections (the five independent X variables) were included to the model to approximate hypothetical yields in future yields using the NEX-DCP30 dataset in the RCP4.5 and RCP8.5 scenarios (Fig. 3). Included in Fig. 3 is yield according to the maximum, minimum and average model temperatures returned by the 32 model runs plus one ensemble mean that make up the NEX-DCP30 dataset. This dataset explores potential yields in a climate where the included variables appear to become more favorable for grape production in Michigan. As a downscaled projection, the NEX-DCP30 data should not be viewed as predictions for each exact year, rather it is the long-term trend from which analysis should be done. It is apparent that yields are likely to increase due to the expected change in Michigan’s climate in the coming decades.

### Michigan’s future growing conditions

**Fig. 4:**
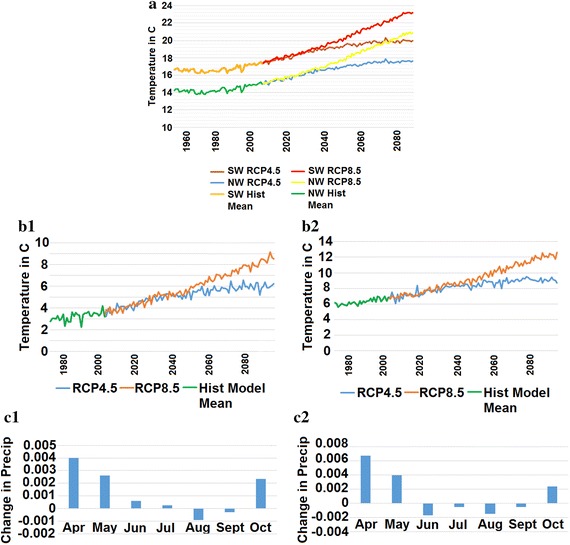
Average growing season temperature (in °C) between southwest and northwest Michigan from 1950 to 2099 according to the mean NEX-DCP30 model outputs (**a**) and average model temperature minimums in April (**b1**) and October (**b2**) and the monthly precipitation change (in in./day × 100) for the RCP 4.5 (**c1**) and RCP 8.5 (**c2**)

As previously mentioned, there are three primary concerns for Michigan’s *vinifera* production. Those include growing season low temperatures, a growing season that is not long enough (from spring to fall frost) and a disadvantageous monthly rainfall distribution. However, according to potential future projections, these issues are likely to become less of a factor in Michigan’s future *vinifera* production. Figure 4a shows the average GSTs for southwest and northwest Michigan. The average temperatures for both regions in both the RCP4.5 and RCP8.5 all show considerable warming out to the end of the twenty-first century. For the RCP4.5 scenario, there is an increase of approximately 2.5 °C in the southwest, and of about 2.75 °C increase in the northwest. In the RCP8.5 scenario, the warming is even clearer. In this scenario, the southwest experiences temperatures nearly 5.5 °C warmer than previous decades, and the northwest registers warming of nearly 6 °C. These warmer temperatures, regardless of scenario or location, would almost certainly change the landscape of Michigan’s *vinifera* industry.

Temperature is not the only climate variable to likely undergo change in the coming decades. Season length is very likely to continue to increase, just as it has for the past decades. This can be seen in the average low temperatures for April and October in southwest Michigan (Fig. 4b1, b2). It should be noted that the trends are near identical for southwest and northwest Michigan, thus the northwest data is not included in the graph. Low temperatures in these critical months (the beginning and end of the growing season, respectively) are going to rise substantially. Average lows in April will rise somewhere between approximately 2.75 and 5 °C (Fig. 4b1). A similar trend is reported in October (Fig. 4b2), with the rise in average monthly minimum temperature between 2.5 and 5 °C. This rise in temperatures has implications for the length of the growing season. Jones and Davis (2000) discuss what these implications looked like in Bordeaux. Earlier budburst, shorter bloom periods and fruit reaching harvest maturity earlier, were a few of the effects of an overall warmer climate over several decades. Similar effects were also seen in Nemani et al. (2001), Malheiro et al. (2013) and Schultze et al. (2014). In these months, average higher minimum temperatures are likely to account for a longer growing season.

Lastly, Michigan’s monthly precipitation distribution is likely to undergo a change that could be beneficial to the *vinifera* industry. Currently, Michigan gets a small amount of rain in the early months of the growing season and too much rain at the end of the season. However, while Michigan is likely to receive a slight increase in annual precipitation (the increase according model means from NEX-DCP30 is not statistically significant), the distribution of precipitation across each month is likely to change. The changes over time for southwest Michigan are reported in Fig. 4c1, c2. In both scenarios, we see more rainfall in the months of April and May, and less rainfall in August and September, which is critical for reducing issues such as fruit rot or other diseases during the critical verasion phenological stage and at harvest. Less precipitation in the end months may also lead to less dilution of sugar (Brix) suspended sugars in individual berries (Gladstones 1992).

## Discussion

### Climate trends

It is clear from the results that the NEX-DCP30 downscaled version of the CMIP5 suite of climate models envisions a different climate for Michigan during the growing season. The climate change has already been occurring over the past few decades (Table 1), and, the changes will continue and even accelerate (Figs. 3, 4). The direct potential changes to grapes growing are shown in Fig. 3, with the multi-linear regression for potential yields into the future. This model was created using yield data from juice grapes (*V. labrusca*) and thus is applicable only to this species. This is because *labrusca* vines respond differently to the same climate conditions compared to other grape varieties. However, while all grape varieties respond differently to climate, general trends can be found applying generally to grapes. Bindi et al. (1997), Jones and Davis (2000) and Nemani et al. (2001) are examples of showing what happens to grape varieties under changing climate scenarios. In Michigan, changes to phenology to *vinifera* and *labrusca* grapes include earlier budburst and later first fall frost events thus creating a longer growing season. While this model is limited to one variety, the trends should still be interesting from a scientific standpoint; namely the increase in yields as a response to a more favorable climate in Michigan. There is also uncertainty in the climate models used in this study, which makes the exact prediction of climate in a particular year very difficult. However, while the predicted year-to-year yields should not be taken literally, the trends are more valuable for analysis. Thus, it is the trend that is substantial, and in the RCP4.5 and RCP8.5 scenarios we see yields reach new highs as conditions become better for grapes.

**Table 1:** Table of variables used in regression model for yield data in southwest Michigan along with equations and accuracy of regressions for frost and GDD variables

Variable	Source	Equation	R^2^
GST	Historical model runs	Tavg of April–October months	
PPT	Historical model runs	Total precipitation of April–October months	
Frost	Regression	−10.410 + (1.389 × MayTmin)	0.52*
GDD	Regression, base 10 °C	−996.347 + (118.021 × JunTavg) + (237.501 × JulTavg) − (298.855 × AugTavg) + (67.267 × SepTavg)	0.61*
eGDD	Historical model run, base 10 °C	Tavg of April	

* Significant at p < .001

The RCP4.5 (Fig. 3a) scenario shows yields eventually leveling off and perhaps even marginally decreasing by the end of the century. This is due to temperatures beginning to drop by a small margin at the end of the twenty-first century and because of the slight increase in precipitation and the potential for frost still being existent (although not as strong as it currently is). RCP8.5 (Fig. 3b) shows a trend that continually increases. This is not likely to be a linear relationship, as there is likely to be a point in the climate where grape growth is slowed. However, the trend of increased production is logical, as temperatures in Michigan eventually become analogous to the temperatures currently seen in other viticultural areas such as California. It should also be noted that this model extrapolates “potential yields.” Pests and diseases, management practices and economics were not included in the model. The goal was only to show specifically what a future climate in Michigan could look like and what the response in the vines might be.

Michigan’s future climate is likely to accommodate *vinifera* production in a better way than currently exists. This research addressed the three major concerns for Michigan’s production (too cool, too short of a season and too much late season rain), and showed that the three issues are likely to lessen in severity to different degrees over time. Michigan, like the rest of the planet, is likely to continue to warm up and thus Michigan’s average GST is likely to increase. Figure 4a displays just how much the GSTs could rise, and if those numbers are even near correct, *vinifera* production in Michigan will be vastly different than it currently is in terms of total acreage and varieties grown.

Growing season length and monthly precipitation distributions are also likely to change as well. This will make a warmer Michigan even more accommodating to *vinifera* production. The authors attempted to create a predictive model for season length, but failed to find a statistical model worth sharing. However, the authors feel that using monthly low temperatures for April and October are a logical analog for showing that Michigan’s growing season is likely to continue to grow beyond the 28.8 day increase discussed in Schultze et al. (2014). A longer season, overall, will get Michigan to the needed 180 days growing season for *vinifera* production. While it is difficult to approximate an exact year for when Michigan will reliably accumulate 180 growing season days, it is reasonable to assume this will occur in the coming decades.

Viticulture in Michigan is limited by precipitation events at the end of the season often evidenced by harvest season cluster-rot, poor ripening and reduced fruit technological maturity. Economically important wine grape varieties possess varying degrees of susceptibility to harvest season cluster rot. However, most of the *V. vinifera* cultivar planted in Michigan are particularly susceptible to cluster rot and they are signature varieties for the Michigan grape and wine industry. The changes in rain events projected by this research (Fig. 4c1, c2) are beneficial for the grape industry, especially the reduction in total rainfall in the final months of the growing season, reducing the potential of detrimental effects of grape quality art harvest.

### Michigan and the future

Michigan’s presence in a “zone of transition” for grapes is a result of temperatures warming over the past decades. The rate of warming is commensurate with the warming seen in most regions of the northern hemisphere since 1980. Prior to 1970, Michigan’s grape producing areas were primarily concentrated on *V. labrusca*, a North American variety. However, *V. vinifera* production has continued to grow from effectively zero in 1970 to >1500 acres in 2011, with a near 300 % increase in acreage from 2000 to 2011 (USDA-NASS 2012). This growth in *vinifera* acreage is likely to continue, as conditions get better for accommodating the varieties of grapes that make traditional wines. Michigan’s location in a “zone of transition” is an analog to climates that have gained and lost the ability to grow grapes thanks to the Medieval Warm Period and ensuing Little Ice Age. These areas include southern England and the Baltic Sea coastline (Pfister 1988; Jones et al. 2005). Jones et al. (2005) discussed the poleward migration of the 12–22 °C isotherm, which is the recommended temperature range for *vinifera* production. Michigan is located within that isotherm and as that area continues to move pole ward, more reliable warm temperatures during the growing season will affect Michigan’s potential for different grape varieties.

**Fig. 5:**
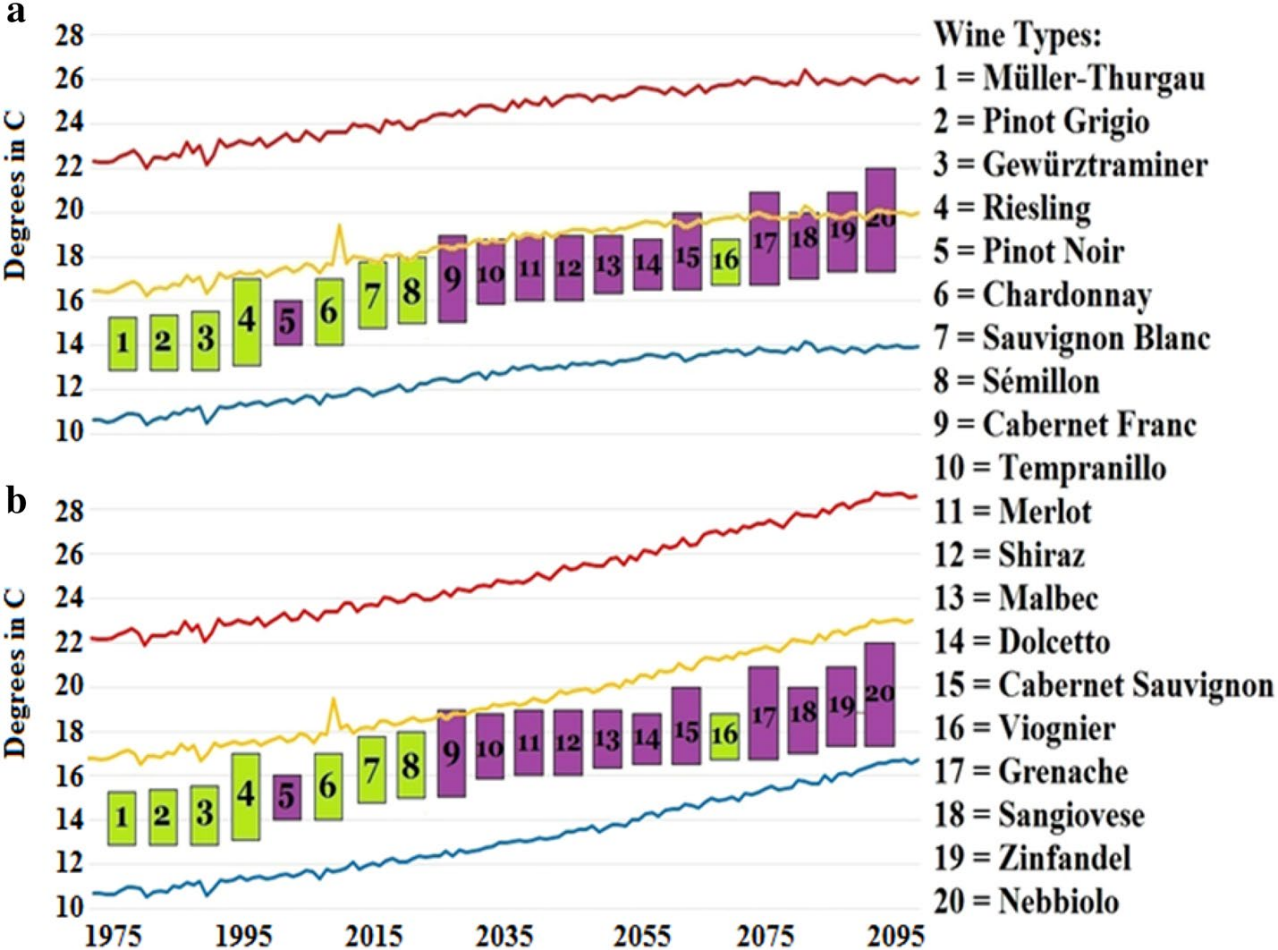
Twenty popular *V. vinifera* varieties (*green* white wine, *purple* red wine) with their requisite growing season temperature thresholds (Jones and Goodrich 2007) along with the RCP4.5 (**a**) and RCP8.5 (**b**) temperature maximum (*red*), average (*yellow*) and minimums (*blue*) from 1975 to 2099

If Michigan’s climate continues to become more promising for *vinifera* production, then it is logical that new varieties should be able to be grown favorably in Michigan. Figure 5 shows different *vinifera* varieties with their approximate optimal temperature thresholds derived from Jones and Goodrich (2007) along with the temperatures predicted by the NEX-DCP30 models for the RCP4.5 and RCP8.5 scenarios. This is meant purely as a hypothetical situation based on temperature, but the figure shows that as time goes on in the twenty-first century, Michigan is likely to be much more viable for different grape varieties. Not all of the varieties are necessarily feasible for production in Michigan (some require a much drier climate), but the point of Fig. 5 is to show that over time, this region should be able to take on more *vinifera* varieties than are currently grown. One can see that in both scenarios, Michigan’s future temperatures are likely going to be able to accommodate warmer varieties, particularly warmer red winegrapes.

Michigan is not the only place that is in a “zone of transition.” Areas at altitude in the western United States and central Europe and areas in southern Russia, northern Germany, southern England and even the Baltic Coast are likely to currently or soon will be in a “zone of transition.” However, Michigan has been growing *vinifera* grapes for several decades. This region can be considered a snapshot of what a “zone of transition” looks like. The trials and errors of this region can serve as a model to the other areas that are soon to gain the ability to grow *vinifera* grapes.

## Conclusion

Michigan’s changing climate has made the region more viable for production of *vinifera* grapes in the past few decades. The state has gone from effectively zero percent of acreage planted as *vinifera* to nearly 15 % of approximately 15,000 acres since 1970 (USDA-NASS 2012). This trend in acreage growth is almost certainly going to continue in the coming decades. The three primary concerns for Michigan’s climate are not likely to be problems of the same magnitude that they once were. The growing season will be considerably warmer and longer, and precipitation is likely to fall at more advantageous times, and less at disadvantageous times. In combination, these changes will continue to shift the landscape as they have been for the past decades.

Data availability was of slight concern for this paper. We did not have yield data for the northwest portion of the state, and there is significant production in that region. The yield regression is based solely on *V. labrusca* grapes in the southwest part of the state. Different varieties respond uniquely to even slight changes in climate. However, we assume that the *labrusca* data is a viable analog for *vinifera* data, as we are looking for the overall future trend, and not an exact number for yield in an exact year. Another concern is the monthly time-step for the NEXDCP-30 dataset. The use of downscaled climate model data introduced uncertainty to the study, as did the use of monthly time-step data, rather than daily data. Uncertainty in this paper, as in any applied climate model study, must be considered as an issue. However, the use of the NEXDCP-30 dataset was still invaluable in this study, as downscaled data below 100 km per pixel made this study possible. The authors also conclude that exploring the future trends for Michigan’s climate with respect to the three primary concerns shows that Michigan’s evolving climate is likely to be generally better for most varieties of grape. It is a near certainty that Michigan will not need to be classified as a “cool” climate for viticulture, and that yields will likely continue to increase as the climate becomes more viable over time and new varieties are introduced to the region.
